# Supplementary material for: An atlas of the aging lung mapped by single cell transcriptomics and deep tissue proteomics
Source: Nat Commun. 2019 Feb 27;10:963. doi: 10.1038/s41467-019-08831-9 (PMC6393476; doi:10.1038/s41467-019-08831-9)
Supplement: Supplementary file 1 — Supplementary Information [file 41467_2019_8831_MOESM1_ESM.pdf]

An atlas of the aging lung mapped by single cell transcriptomics and deep tissue proteomics

Ilias Angelidis[1*], Lukas M. Simon[2*], Isis E. Fernandez[1], Maximilian Strunz[1], Christoph H. Mayr[1], Flavia R. Greiffo[1], George Tsitsiridis[2], Meshal Ansari[1, 2], Elisabeth Graf[3], Tim-Matthias Strom[3], Monica Nagendran[4], Tushar Desai[4], Oliver Eickelberg[5], Matthias Mann[6], Fabian J. Theis[2,7, #] ,and Herbert B. Schiller[1, #]

1. Helmholtz Zentrum München, Institute of Lung Biology and Disease, Member of the German Center for Lung Research (DZL), Munich, Germany
2. Helmholtz Zentrum München, Institute of Computational Biology, Munich, Germany
3. Helmholtz Zentrum München, Institute of Human Genetics, Munich, Germany
4. Department of Internal Medicine, Division of Pulmonary and Critical Care, Stanford University School of Medicine, Institute for Stem Cell Biology and Regenerative Medicine, Stanford University School of Medicine, Stanford, United States of America
5. University of Colorado, Department of Medicine, Division of Respiratory Sciences and Critical Care Medicine, Denver, CO, USA
6. Max Planck Institute of Biochemistry, Department of Proteomics and Signal Transduction, Martinsried, Germany
7. Department of Mathematics, Technische Universität München, Munich, Germany

*… these authors contributed equally to this work
... correspondence to Fabian J. Theis (fabian.theis@helmholtz-muenchen.de)
and Herbert B. Schiller (herbert.schiller@helmholtz-muenchen.de)

Supplementary Figures

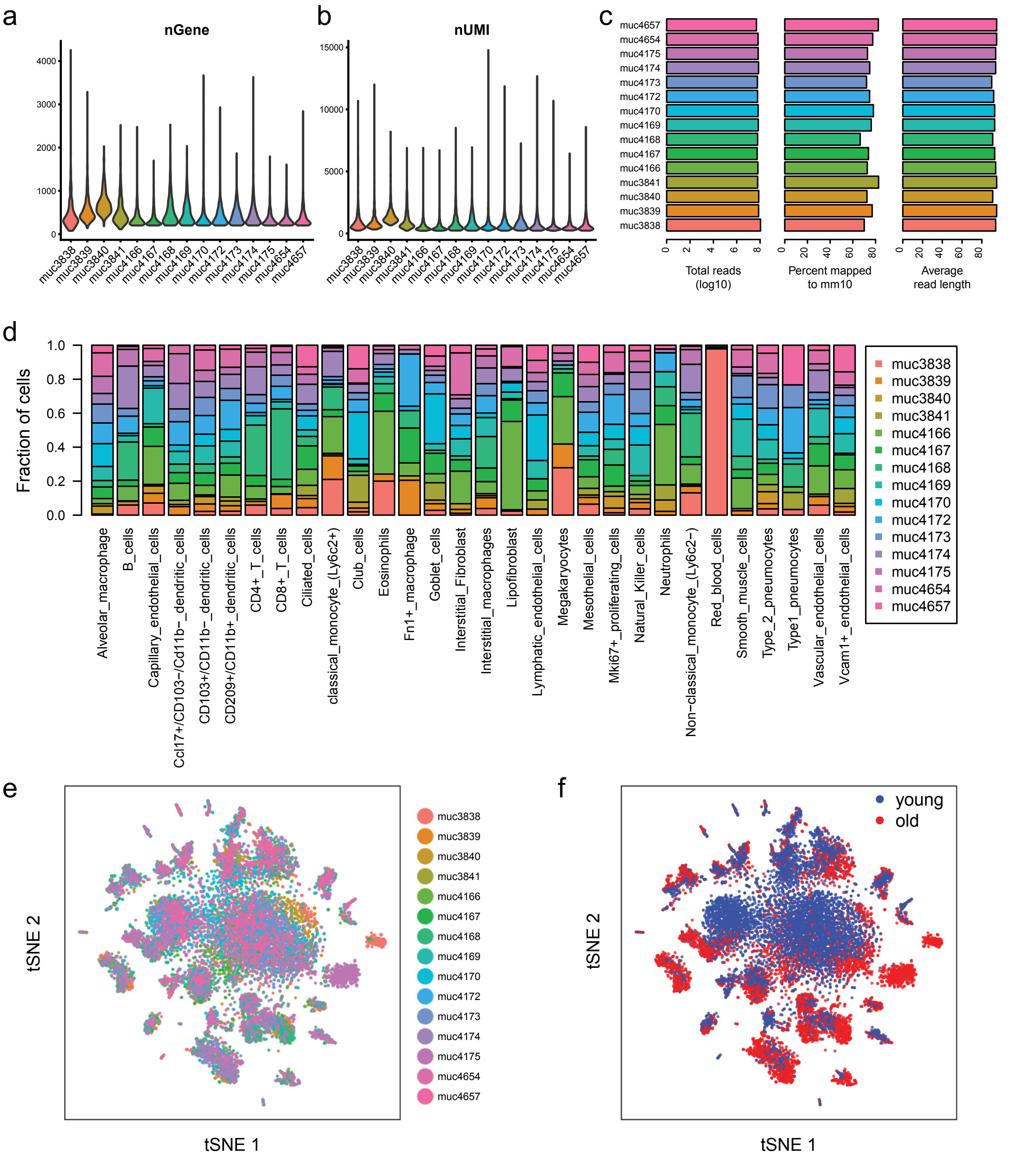

Supplementary Figure 1. High technical reproducibility enables integration of the 15 mouse experiments. (**a, b**) The violin plots show the distrib-ution of the (**a**) number of genes detected per cell and (**b**) total UMI counts per cell across mice, respectively. (**c**) scRNA-seq alignment statistics show comparable values across mice. (**d**) Cell type identity and the fraction of cells per mouse are shown on the X and Y axes respectively. (**e, f**) tSNE visualization colored by (**e**) mouse sample and (**f**) age group.

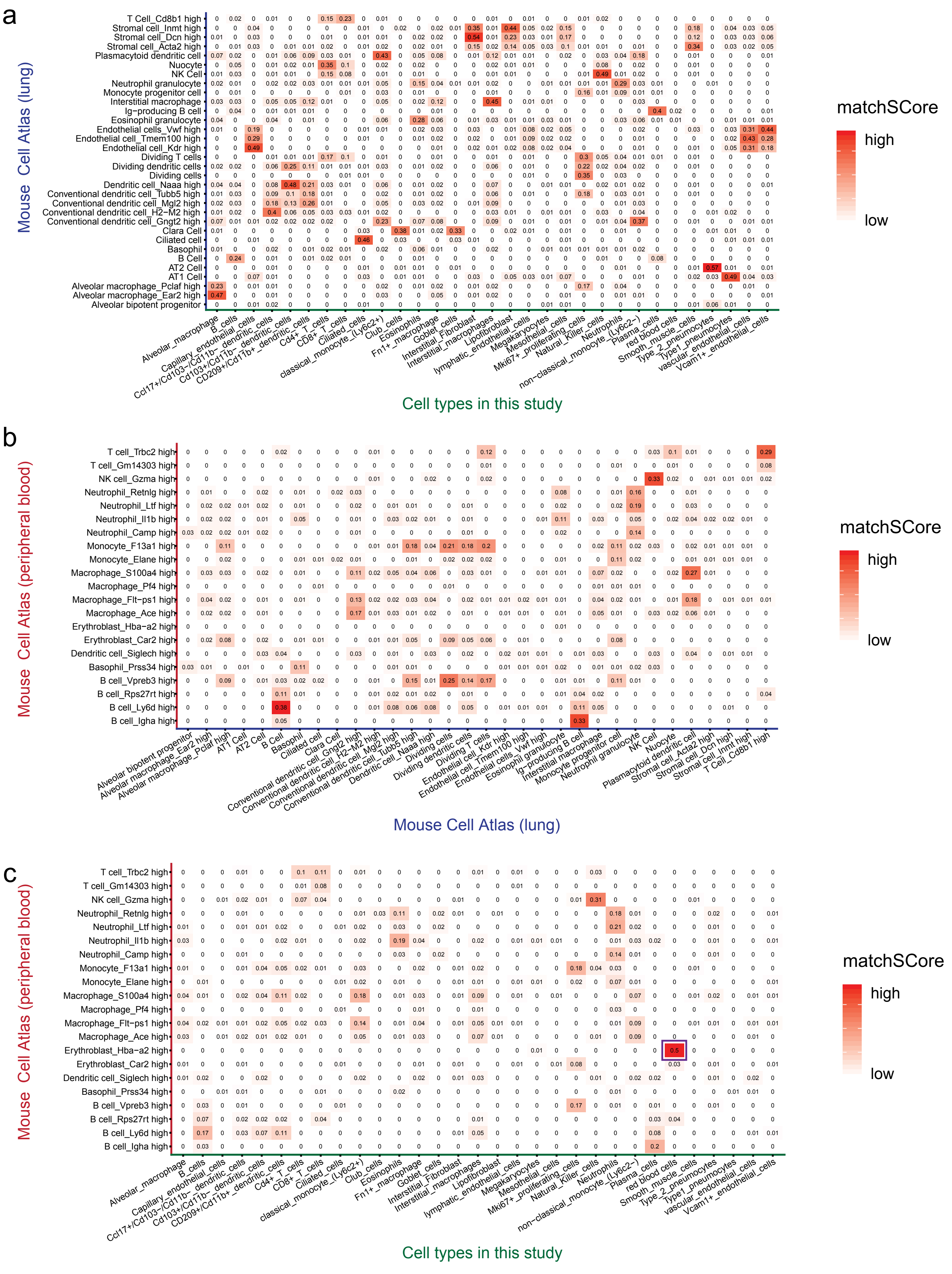

a
Mouse Cell Atlas (lung)
Cell types in this study
matchSCore
high
low
b
Mouse Cell Atlas (peripheral blood)
Mouse Cell Atlas (lung)
matchSCore
high
low
c
Mouse Cell Atlas (peripheral blood)
Cell types in this study
matchSCore
high
low

d

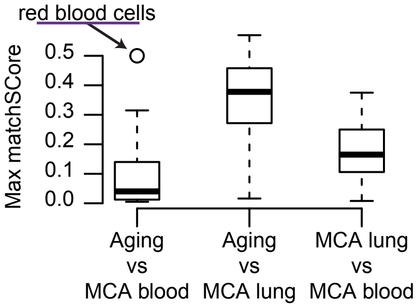

Supplementary Figure 2. Comparison with the Mouse Cell Atlas validates lung cell identities. (**a-c**) The matchSC score comparison between the clusters in this study, the MCA lung and peripheral blood signatures is shown. Red and white colors indicate high and low matchSC scores, respectively. The outlier in panel c represents red blood cells (purple rectangle). (**d**) The box plot shows the distribution of maximal matchSC scores for each cluster across the comparisons between these three data sets. The box represents the inter-quartile range, the horizontal line in the box is the median, and the whiskers represent 1.5 times the inter-quartile range. The outlier in the comparison between cell types in this study and the MCA blood data represents red blood cells (underlined in purple).

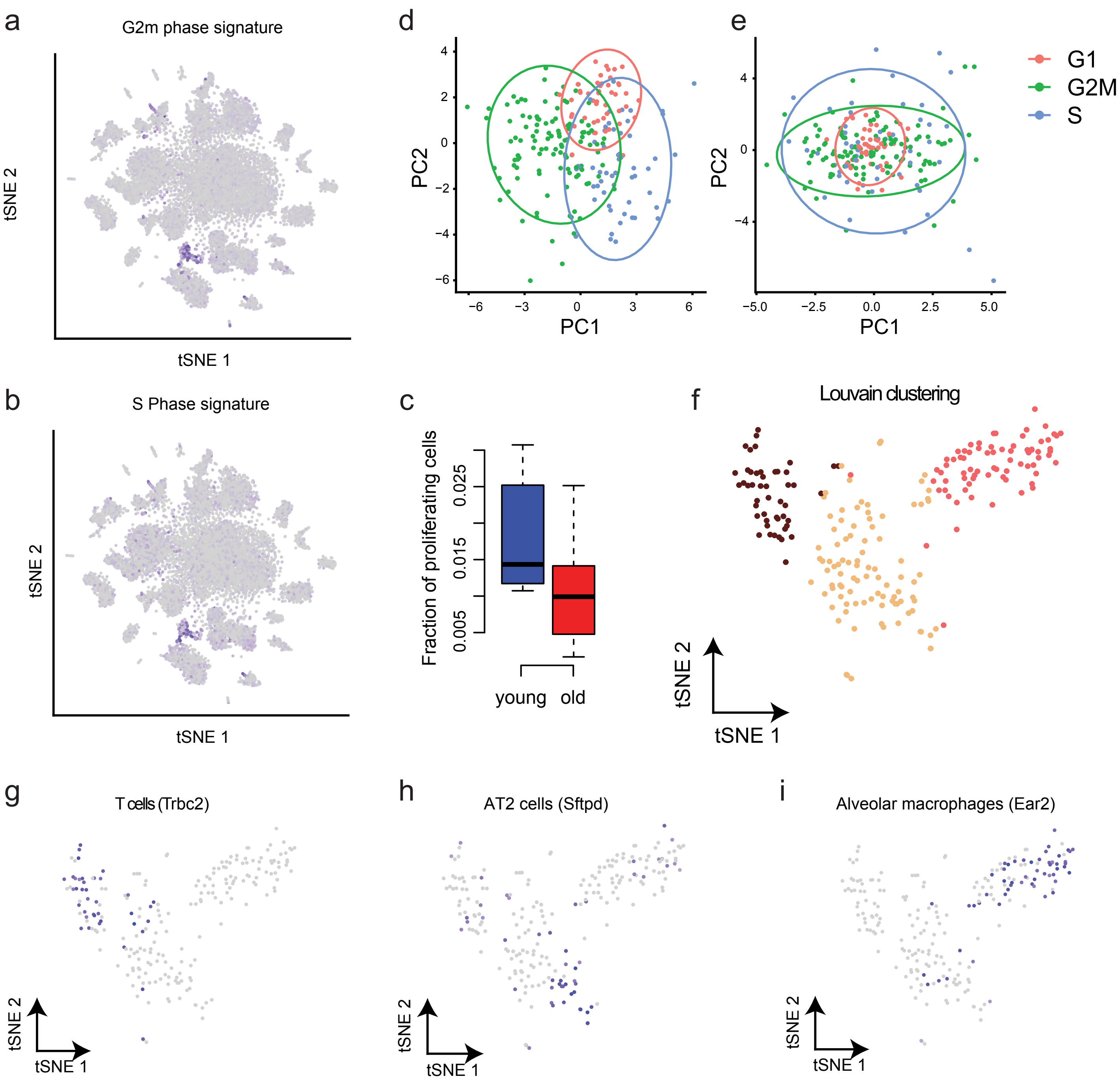

Supplementary Figure 3. Cell-cycle analysis reveals reduced proliferative capacity of T cells, Alveolar macrophages and Type-2 pneumocytes in aged lungs. (**a, b**) The `Mki67+ proliferating cell´ cluster (Fig. 1) showed high expression of (**a**) G2M- and (**b**) S-phase cell cycle signatures. (**c**) A higher fraction of proliferating cells was observed in young (n = 8 animals) compared to old (n = 7 animals) mice. The box represents the interquartile range, the horizontal line in the box is the median, and the whiskers represent 1.5 times the interquartile range. (**d**) PCA based on cell cycle marker genes revealed clustering by cell cycle phase and (**e**) the removal of this effect after regressing out the cell cycle effect. Cells are colored by cell cycle phase as assigned by Seurat. (**f**) Unsupervised Louvain clustering revealed three distinct cell clusters. (**g-i**) tSNE visualization colored by the expression of cell type marker genes (**g**) Trbc2, (**h**) Sftpd and (**i**) Ear2 corresponding to T cells, Type 2 pneumocytes and alveolar macrophages, respectively.

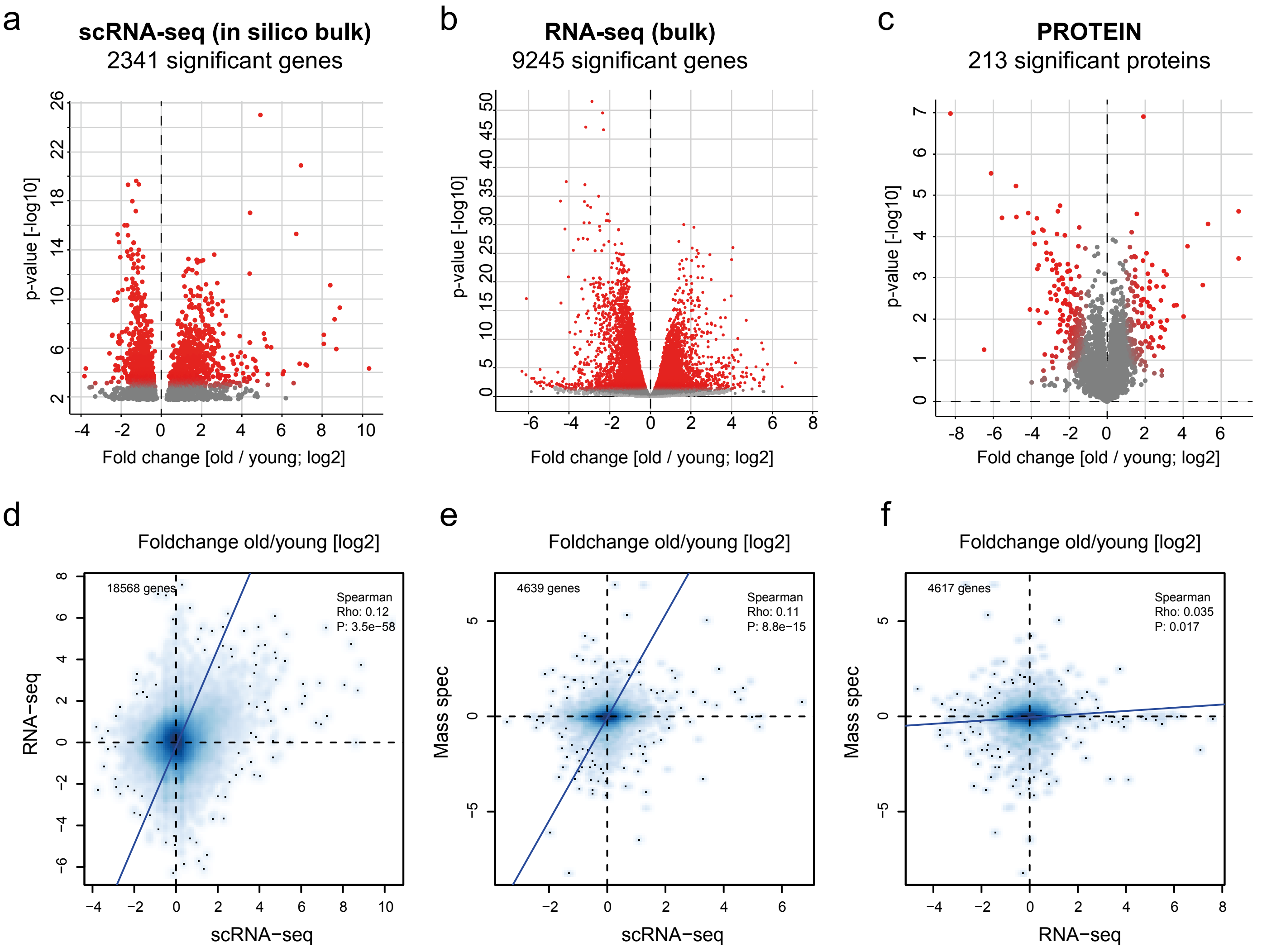

Supplementary Figure 4. Multi-omics lung aging data displays significant correspondence. Volcano plots show the significantly regulated genes from (**a**) in from scRNA-seq, (**b**) bulk RNA-seq and (**c**) mass spectrometry. (**d-f**) Differential expression results from multi-omics experiments show significant correspondence. X and Y axes illustrate the log2 fold changes calculated from the (**d**) RNA-seq and scRNA-seq (in silico bulk) experiments, (**e**) the mass spectrometry (protein) and scRNA-seq (in silico bulk) experiments, and (**f**) the mass spectrometry (protein) and RNA-seq experiments. Blue line indicates the Deming regression fit. Black dotted horizontal and vertical lines indicate 0 values (no differential expression) for the in silico bulk and protein data, respectively.

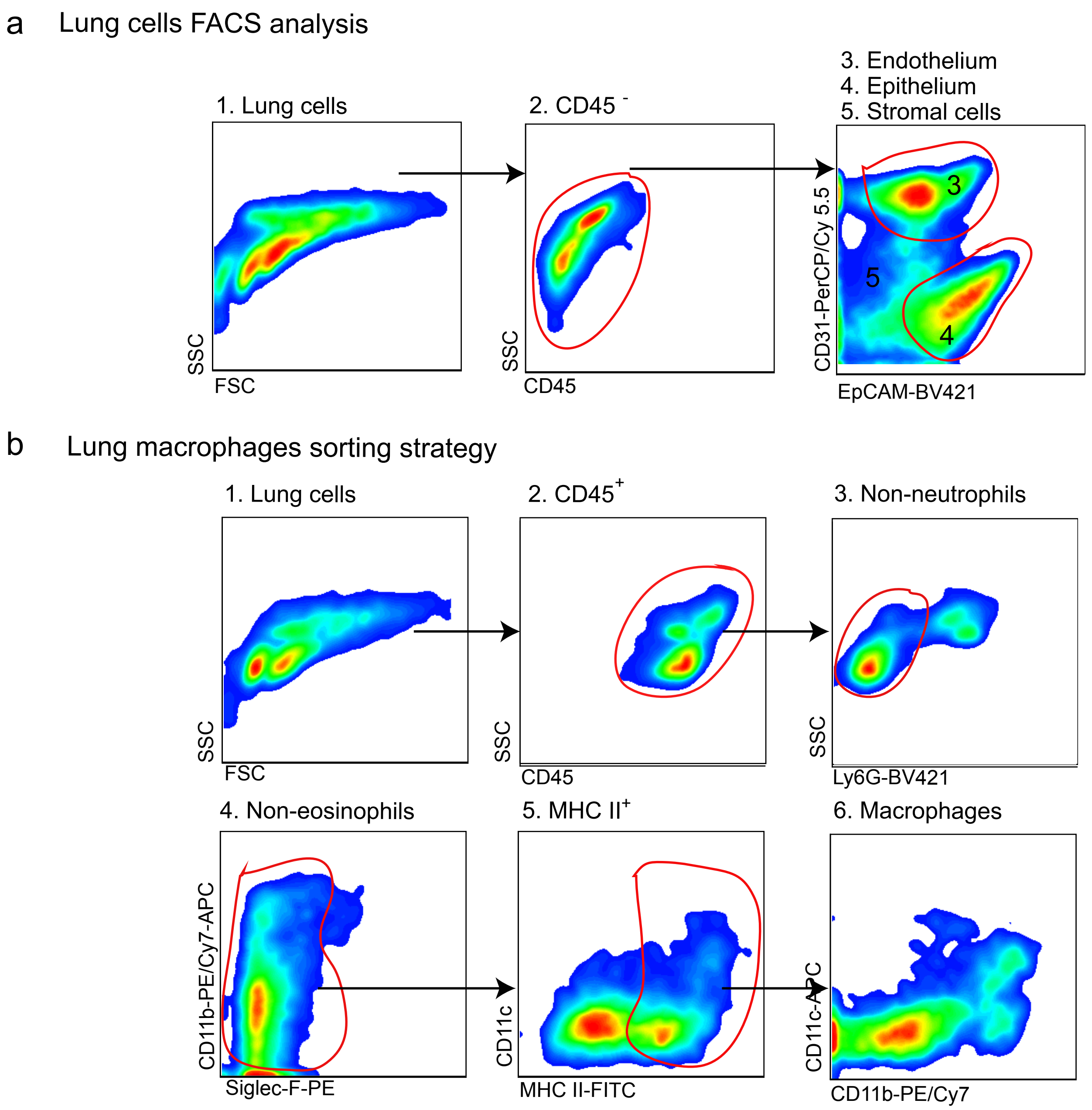

Supplementary Figure 5. scRNA-seq data validation using bulk RNA-seq of flow sorted cell populations from young and old mice. Cells were sorted by using the (**a**) CD45 negative fraction of the cell isolate stained for anti-mouse CD31, and EpCAM antibodies. Epithelial cells were sorted as CD31- cells and EpCAM+ cells (**a-4**). For sorting macrophages we used the (**b**) CD45 positive fraction and stained with anti-mouse CD11c, CD11b, MHC II, Siglec-F and Ly6G antibodies. For flow cytometry sorting, neutrophils were excluded by selection of Ly6G negative cells. Macrophages were sorted as MHCII+, CD11c+,CD11b+.

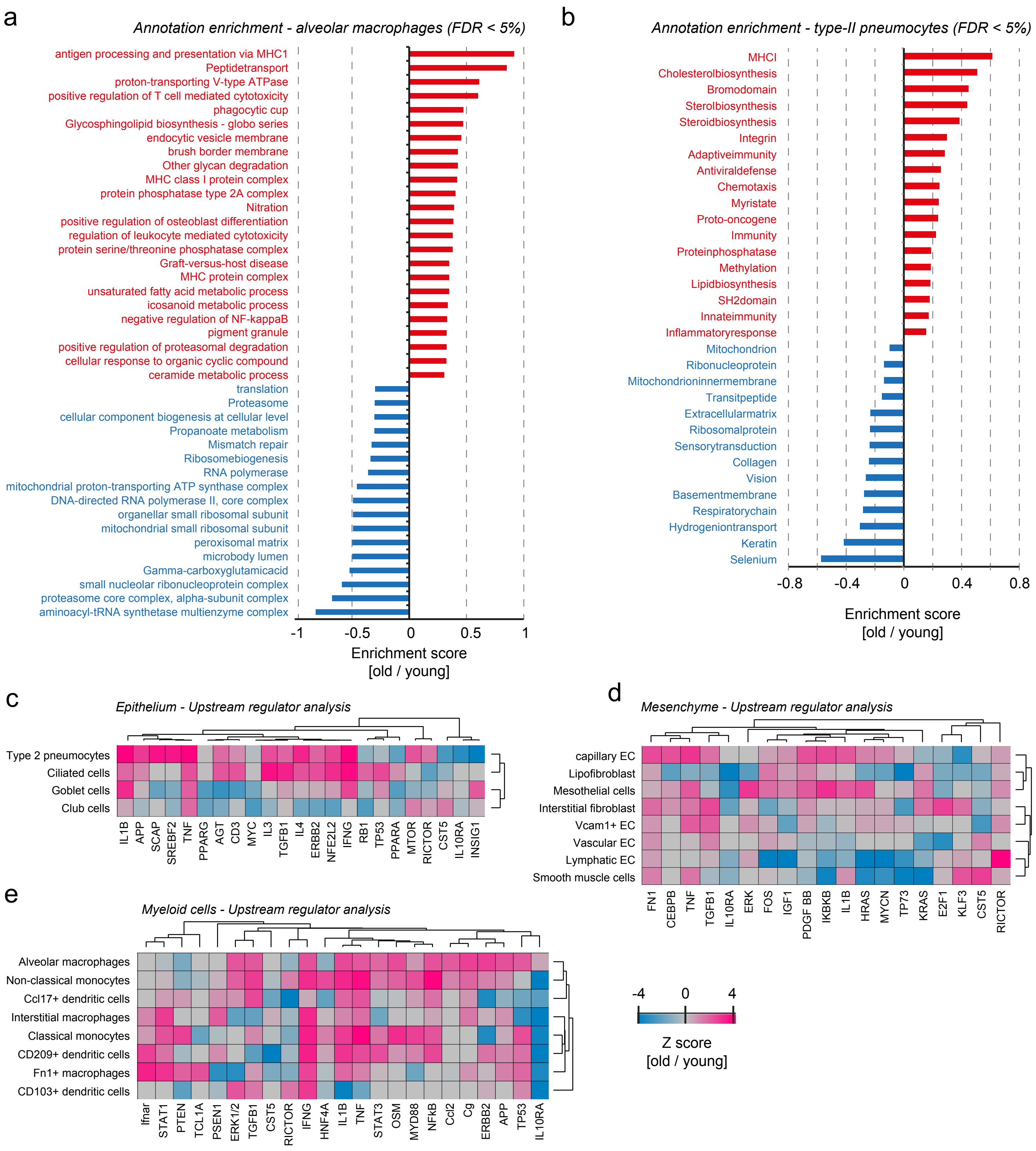

Supplementary Figure 6. Pathway and upstream regulator analysis reveals cell type specific effects of aging. (**a, b**) The bar graph shows the result of a gene annotation enrichment analysis for (**a**) alveolar macrophages, and (**b**) type-II pneumocytes, respectively. Gene categories with positive (upregulated in old) and negative scores (downregulated in old) are highlighted in red and blue respectively. (**c-e**) Upstream regulators are predicted based on the observed gene expression changes for (**c**) epithelial, (**d**) mesenchymal, and (**e**) myeloid cells. Cell types and regulators were grouped by unsupervised hierarchical clustering (Pearson correlation) and the indicated transcriptional regulators and cytokines, growth factors and ECM proteins are color coded based on the activation score as shown.

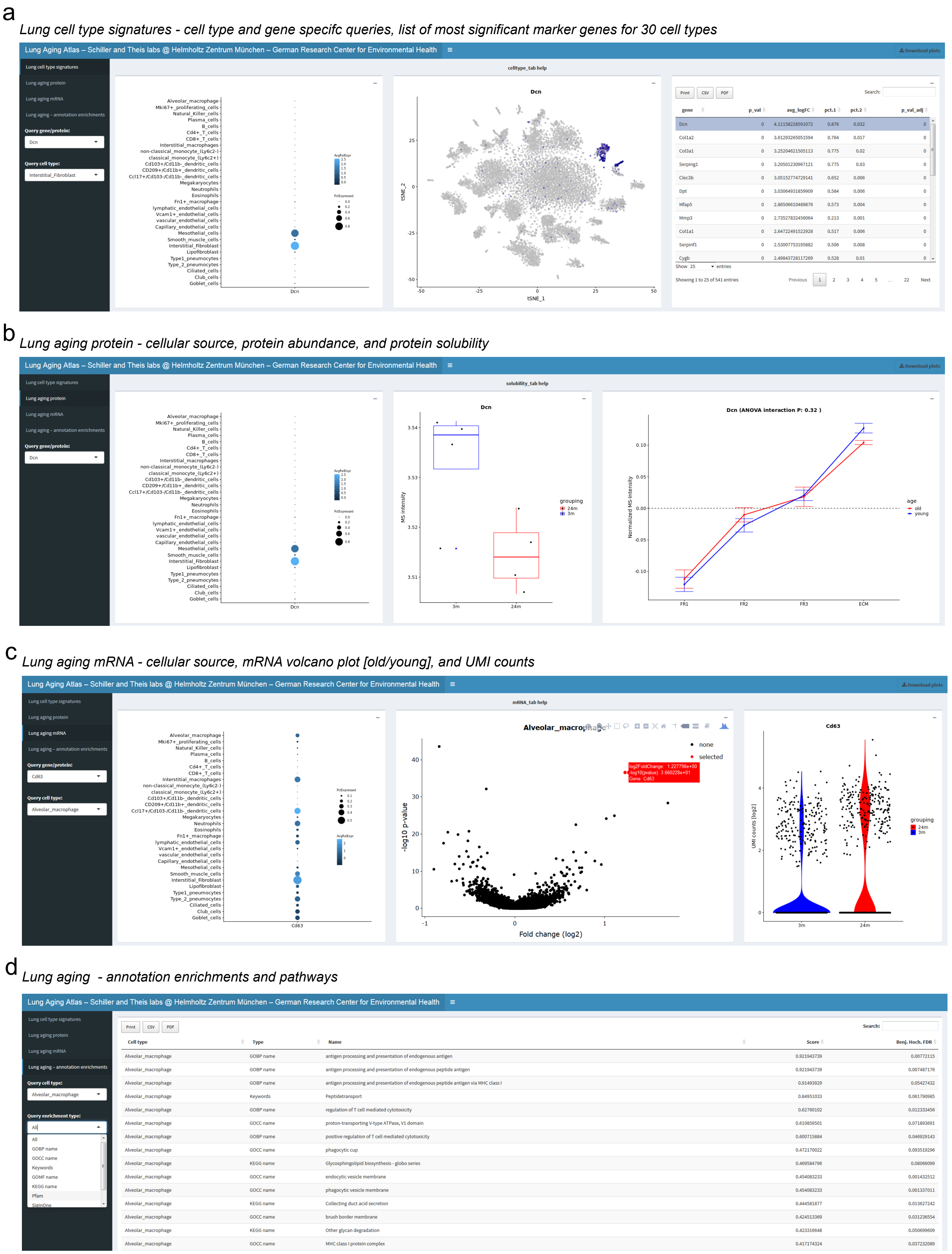

a
Lung cell type signatures - cell type and gene specifc queries, list of most significant marker genes for 30 cell types
Lung Aging Atlas – Schiller and Theis labs @ Helmholtz Zentrum München – German Research Center for Environmental Health
Lung cell type signatures
Lung aging protein
Lung aging mRNA
Lung aging – annotation enrichments
Query gene/protein:
Dcn
Query cell type:
Interstitial_Fibroblast
celltype_tab help
tSNE_1
tSNE_2
Showing 1 to 25 of 541 entries
b
Lung aging protein - cellular source, protein abundance, and protein solubility
solubility_tab help
Dcn (ANOVA interaction P: 0.32)
MS intensity
Normalized MS-intensity
grouping
age
old
young
c
Lung aging mRNA - cellular source, mRNA volcano plot [old/young], and UMI counts
mRNA_tab help
Cd63
Alveolar_macrophage
Fold change (log2)
-log10 p-value
UMI counts [log2]
none
selected
d
Lung aging - annotation enrichments and pathways
Query cell type:
Alveolar_macrophage
Query enrichment type:
Cell type
Type
Name
Score
Benj. Hoch. FDR

Supplementary Figure 7. A user friendly and interactive webtool enables navigating the Lung Aging Atlas. (**a**) The first tab `Lung cell type signatures´ provides a cell type dotplot (left panel) and a color coded tSNE map (middle panel) for gene specific queries and illustrates cell type specific expression of any gene of interest. A cell type query produces a list of top marker genes for the cell type of interest (right panel). (**b**) The panel `Lung aging protein´ features a dot plot to illustrate the most likely cellular source of the protein of interest (left panel), a box plot to show alterations in total lung tissue protein abundance in old mice (middle panel), and a line plot to show protein solubilities. Protein solubility is measured by relative quantification of protein abundance across four fractions. Fraction 1 (FR1) contains proteins with highest and fraction 4 (ECM) with lowest solubility. Curves that peak on the right (ECM) thus represent insoluble proteins. (**c**) The tab `Lung aging mRNA´ again features the dotplot (left panel), a volcano plot that shows fold changes [old/young] on the x-axis and -log1 p-values on the y-axis (middle panel), and a violin plot of the log2 UMI counts illustrating mRNA abundance in young and old mice. The dot and violin plot are navigated with the gene specific query, while the volcano plot requires navigation via the cell type query. The volcano plot has a toggle over function that allows identification of genes and can thus be used to browse through differential gene expression between young and old cells of any cell type of interest. (**d**) In the tab `Lung aging - annotation enrichments´, the gene annotation enrichments between old and young can be browsed for all 3 cell types.
