# Supplementary material for: An atlas of the aging lung mapped by single cell transcriptomics and deep tissue proteomics
Source: Nat Commun. 2019 Feb 27;10:963. doi: 10.1038/s41467-019-08831-9 (PMC6393476; doi:10.1038/s41467-019-08831-9)
Supplement: Supplementary file 3 — Description of Additional Supplementary Files [file 41467_2019_8831_MOESM3_ESM.docx]

**Description of Supplementary Files**

**File Name:** **Supplementary Data 1**

**Description:** Differential gene expression analysis to determine cell type specific marker genes. The clusters were annotated with assumed cell type identities based on (1) known marker genes derived from expert annotation in literature, and (2) enrichment analysis using Fisher’s exact test of gene expression signatures of isolated cell types from databases including ImmGen and xCell.

**File Name:** **Supplementary Data 2**

**Description:** (Tab 1) Differential protein abundance in total lung tissue proteomes of young (3 months) and old mice (24 months). (Tab 2) Differential gene expression from in silico bulk samples (derived from the scRNA-seq data by summing expression counts from all cells for each mouse individually). (Tab 3) Differential gene expression in whole lung bulk RNAseq data of three replicates of young (3 months) and old mice (22 months). (Tab 4) Differential gene expression profiles of flow sorted macrophages. (Tab 5) Differential gene expression profiles of flow sorted epithelial cells.

**File Name:** **Supplementary Data 3**

**Description:** Two dimensional annotation enrichment analysis was performed to reveal common or distinct regulation of gene annotation categories in the transcriptome or proteome.

**File Name:** **Supplementary Data 4**

**Description:** Quantitative Detergent Solubility Profiling of whole lung tissue proteomes from young and old mice.

**File Name:** **Supplementary Data 5**

**Description:** Cell type-resolved differential gene expression testing between age groups in the single cell data.

**File Name:** **Supplementary Data 6**

**Description:** Cell type resolved 1D annotation enrichment analysis reveals regulated gene categories [old/young].

**File Name:** **Supplementary Data 7**

**Description:** `Ambient´ mRNA.
