# Supplementary material for: An atlas of the aging lung mapped by single cell transcriptomics and deep tissue proteomics
Source: Nat Commun. 2019 Feb 27;10:963. doi: 10.1038/s41467-019-08831-9 (PMC6393476; doi:10.1038/s41467-019-08831-9)
Supplement: Supplementary file 11 — Reporting Summary [file 41467_2019_8831_MOESM11_ESM.pdf]

natureresearch

Corresponding author(s): Herbert B. Schiller

Last updated by author(s): Jan 17, 2019

Reporting Summary

Nature Research wishes to improve the reproducibility of the work that we publish. This form provides structure for consistency and transparency in reporting. For further information on Nature Research policies, see Authors & Referees and the Editorial Policy Checklist.

Statistics

For all statistical analyses, confirm that the following items are present in the figure legend, table legend, main text, or Methods section.

n/a	Confirmed	
☐	☒	The exact sample size (*n*) for each experimental group/condition, given as a discrete number and unit of measurement
☐	☒	A statement on whether measurements were taken from distinct samples or whether the same sample was measured repeatedly
☐	☒	The statistical test(s) used AND whether they are one- or two-sided<br>*Only common tests should be described solely by name; describe more complex techniques in the Methods section.*
☐	☒	A description of all covariates tested
☐	☒	A description of any assumptions or corrections, such as tests of normality and adjustment for multiple comparisons
☐	☒	A full description of the statistical parameters including central tendency (e.g. means) or other basic estimates (e.g. regression coefficient) AND variation (e.g. standard deviation) or associated estimates of uncertainty (e.g. confidence intervals)
☐	☒	For null hypothesis testing, the test statistic (e.g. *F*, *t*, *r*) with confidence intervals, effect sizes, degrees of freedom and *P* value noted<br>*Give P values as exact values whenever suitable.*
☐	☒	For Bayesian analysis, information on the choice of priors and Markov chain Monte Carlo settings
☐	☒	For hierarchical and complex designs, identification of the appropriate level for tests and full reporting of outcomes
☐	☒	Estimates of effect sizes (e.g. Cohen's *d*, Pearson's *r*), indicating how they were calculated

Our web collection on statistics for biologists contains articles on many of the points above.

Software and code

Policy information about availability of computer code

Data collection	The Drop-seq pipeline (https://github.com/broadinstitute/Drop-seq) was used to generate UMI count matrices. The MaxQuant (https://www.biochem.mpg.de/5111795/maxquant) software was used to process proteomics data.
Data analysis	Custom R code was used and has been deposited on GitHub (https://github.com/theislab/2018_Angelidis). Perseus was used (https://www.biochem.mpg.de/5111810/perseus). The commercial software Ingenuity Pathway Analysis was used (https://www.qiagenbioinformatics.com/products/ingenuity-pathway-analysis).

For manuscripts utilizing custom algorithms or software that are central to the research but not yet described in published literature, software must be made available to editors/reviewers. We strongly encourage code deposition in a community repository (e.g. GitHub). See the Nature Research guidelines for submitting code & software for further information.

Data

Policy information about availability of data

All manuscripts must include a data availability statement. This statement should provide the following information, where applicable:

- Accession codes, unique identifiers, or web links for publicly available datasets
- A list of figures that have associated raw data
- A description of any restrictions on data availability

Proteome raw data can be downloaded from the PRIDE repository under the accession number PXD012307[http://www.ebi.ac.uk/pride/archive/projects/PXD012307]. scRNA-seq, whole lung tissue bulk and flow-sorted cell populations bulk raw data can be downloaded from the Gene Expression Omnibus under the accession number GSE124872[https://www.ncbi.nlm.nih.gov/geo/query/acc.cgi?acc=GSE124872]. The whole lung aging atlas can be accessed via an interactive user-friendly webtool at: https://theislab.github.io/LungAgingAtlas. A reporting summary for this Article is available as a Supplementary Information file. All other data supporting the findings of this study are available from the corresponding authors upon reasonable request.

October 2018

Field-specific reporting

Please select the one below that is the best fit for your research. If you are not sure, read the appropriate sections before making your selection.

☒ Life sciences ☐ Behavioural & social sciences ☐ Ecological, evolutionary & environmental sciences

For a reference copy of the document with all sections, see nature.com/documents/nr-reporting-summary-flat.pdf

Life sciences study design

All studies must disclose on these points even when the disclosure is negative.

Sample size	We sequenced ~1000 single cells per mouse and used 7 and 8 young and old mice respectively, enabling us to do statistics both on single cell and mouse level.
Data exclusions	No data was excluded
Replication	The study contained 7 and 8 mouse replicates per age group. In addition bulk RNAseq data was integrated to validate findings.
Randomization	Young and old mice were indexed and sequenced together randomized on sequencing lanes.
Blinding	Blinding was not possible because the wet lab experiments were done by one single person.

Reporting for specific materials, systems and methods

We require information from authors about some types of materials, experimental systems and methods used in many studies. Here, indicate whether each material, system or method listed is relevant to your study. If you are not sure if a list item applies to your research, read the appropriate section before selecting a response.

Materials & experimental systems

n/a	Involved in the study	
☐	☒	Antibodies
☒	☐	Eukaryotic cell lines
☒	☐	Palaeontology
☐	☒	Animals and other organisms
☒	☐	Human research participants
☒	☐	Clinical data

Methods

n/a	Involved in the study	
☒	☐	ChIP-seq
☐	☒	Flow cytometry
☒	☐	MRI-based neuroimaging

Antibodies

Antibodies used	The following primary (1) and secondary (2) antibodies were used: (1) CC10 rabbit (Santa Cruz, sc-25554, 1:100), Foxj1 mouse (Santa Cruz, sc-53139, 1:50), Collagen IV rabbit (Abcam, ab6586, 1:100), (2) donkey anti-mouse Alexa Fluor (AF) 647 (Invitrogen, A21447), donkey anti-rabbit AF 568 (Invitrogen, A10042), donkey anti-goat AF 488 (Invitrogen, A21202). Counterstain with LipidTox was performed using HCS LipidTOX deep red neutral lipid stain (Invitrogen, H34477, 1:200).
Validation	Only well established antibodies were used.

Animals and other organisms

Policy information about studies involving animals; ARRIVE guidelines recommended for reporting animal research

Laboratory animals	Female and male C57BL/6 mice from different age groups (3, 22, 24 months old) were used.
Wild animals	The study did not involve wild animals.
Field-collected samples	The study did not involve samples collected from the field.
Ethics oversight	Pathogen-free C57BL/6 mice were obtained from Charles River and housed in rooms maintained at constant temperature and humidity with a 12-h light cycle. Animals were allowed food and water ad libitum. For this study, organs were obtained from mice that had to be sacrificed because of excessive breeding. Animal handling was performed according to strict governmental and international guidelines and ethical oversight by the local government for the administrative region of Upper Bavaria, Germany.

Note that full information on the approval of the study protocol must also be provided in the manuscript.

Flow Cytometry

Plots

Confirm that:

☒ The axis labels state the marker and fluorochrome used (e.g. CD4-FITC).

☒ The axis scales are clearly visible. Include numbers along axes only for bottom left plot of group (a 'group' is an analysis of identical markers).

☒ All plots are contour plots with outliers or pseudocolor plots.

☒ A numerical value for number of cells or percentage (with statistics) is provided.

Methodology

Sample preparation	Sample preparation is discribed in detail in the methods section of the manuscript.
Instrument	Data acquisition was performed in a BD Fortessa flow cytometer (Becton Dickinson; Heidelberg, Germany).
Software	Data were analyzed using the FlowJo software (TreeStart Inc; Ashland, OR, USA).
Cell population abundance	2000 macrophages and 2000 epithelial cells were sorted for bulk RNA seq as described in the methods section of the manuscript.
Gating strategy	Cells were sorted by using the CD45 negative fraction of the cell isolate stained for anti-mouse CD31, and EpCAM antibodies. Epithelial cells were sorted as CD31- cells and EpCAM+ cells. For sorting macrophages we used the CD45 positive fraction and stained with anti-mouse CD11c, CD11b, MHC II and Ly6G antibodies. For flow cytometry sorting, neutrophils were excluded by selection of Ly6G negative cells. Macrophages were sorted as MHCII+, CD11c+,CD11b+ as previously described66. Data acquisition was performed in a BD Fortessa flow cytometer (Becton Dickinson; Heidelberg, Germany). All stainings were performed per 300.000 cells in the following dilutions: CD31 (1:300), EpCAM (1:50), H2K1 (1:50), CD11c (1:100), CD11b (1:25), MHCII (1:50), Ly6G (1:10).

☒ Tick this box to confirm that a figure exemplifying the gating strategy is provided in the Supplementary Information.
